# Modelling Behaviour of a Carbon Epoxy Composite Exposed to Fire: Part I—Characterisation of Thermophysical Properties

**DOI:** 10.3390/ma10050494

**Published:** 2017-05-04

**Authors:** Pauline Tranchard, Fabienne Samyn, Sophie Duquesne, Bruno Estèbe, Serge Bourbigot

**Affiliations:** 1 Univ. Lille, UMR 8207, UMET, Unité Matériaux et Transformations, Lille F 59 000, France; pauline.tranchard@univ-lille1.fr (P.T.); fabienne.samyn@ensc-lille.fr (F.S.); sophie.duquesne@ensc-lille.fr (S.D.); 2 Thermal Tech Centre, AIRBUS Operation S.A.S, 316 Route de Bayonne, Toulouse 31060, France; bruno.estebe@airbus.com

**Keywords:** carbon epoxy laminates, anisotropy, high-temperature properties, thermal analysis

## Abstract

Thermophysical properties of a carbon-reinforced epoxy composite laminate (T700/M21 composite for aircraft structures) were evaluated using different innovative characterisation methods. Thermogravimetric Analysis (TGA), Simultaneous Thermal analysis (STA), Laser Flash analysis (LFA), and Fourier Transform Infrared (FTIR) analysis were used for measuring the thermal decomposition, the specific heat capacity, the anisotropic thermal conductivity of the composite, the heats of decomposition and the specific heat capacity of released gases. It permits to get input data to feed a three-dimensional (3D) model given the temperature profile and the mass loss obtained during well-defined fire scenarios (model presented in Part II of this paper). The measurements were optimised to get accurate data. The data also permit to create a public database on an aeronautical carbon fibre/epoxy composite for fire safety engineering.

## 1. Introduction

To limit fire risks in aircraft, aviation authorities apply stringent safety regulations. Specific tests, known as certifications tests, are required to validate the aircraft design. Currently, during the development phase of an aircraft, the numerical simulation is largely used in fire safety engineering for metallic structures. It permits to draw an optimised design and to replace test: it is called “virtual testing”. Composite materials are used in the new aircraft generation as structural materials, and hence, the numerical methodology developed for metallic structure is no longer applicable. It is the reason why the development of new models to characterise the fire behaviour of composites is required. In this context, a recent paper was published on the fire behaviour of a carbon fibre-reinforced epoxy resin (CFRP) plate for aeronautical application [1]. It involved a novel bench scale test equipped with a complete set of instrumentation designed to investigate the fire behaviour of materials. This test is compliant with the ISO2685:1998(E) aeronautical certification test [2]. Temperature profile, mass loss and quantity of released gases can be measured as a function of time: it showed the capability of this test to enhance the understanding of the fire behaviour of CFRP. Physical, thermal and chemical phenomena occurring during this fire test were examined in this work. It took into account a variety of processes including thermal expansion (inducing apparition of cracks), thermal decomposition of the resin, internal pressure phenomenon, gas migration through the material and thermal delamination. All these analyses provided crucial information to validate the methodology developed for modelling the behaviour of a composite laminate exposed to fire. The goal of this work is to develop a comprehensive physical model on the fire behaviour of CFRP. It is a two-part paper: Part I describes and analyses the temperature-dependant thermophysical properties of CFRP (here the T700/M21 composite), while Part II reports a three-dimensional thermochemical model of the fire behaviour of CFRP.

In this paper, methodologies to determine thermophysical properties of a carbon fibre/epoxy composite from ambient up to high temperature and as a function of the decomposition degree are presented. Firstly, experimental and numerical methods reported in the literature to get the physical (density), chemical (kinetic parameters) and thermal (heat capacity and thermal conductivity) parameters of a composite laminate are discussed. As reported in the literature [3,4,5], those parameters are necessary to get a suitable model. Secondly, the methods developed in this work to determine the thermophysical properties of the composite are presented. The resulting parameters are then used as input data for further modelling (discussed in Part II of this paper).

## 2. Review of the Literature

The determination of the thermophysical properties of materials is one of the keys to model the fire behaviour of composite material [6]. These properties are temperature dependent and they are difficult to measure at high temperature (higher than the temperature of decomposition of the material). It is the reason why the authors often considered these properties by extrapolating the results obtained close to ambient temperature to higher temperatures.

The density can be generally written using two methods: (i) as a function of the different constituents of the material; or (ii) as a function of each step of decomposition. Density and mass can be expressed as a function of *α*, the decomposition degree, *V*_f_, the volume fraction of fibre, *V*_r_^0^, the initial resin content, and of the densities of, *ρ*_f_, fibre, *ρ*_r_, resin and *ρ*_g_, gas (Equation (1)) [7].

(1) $$\rho \;=\;V_{f}\rho_{f}\;+\;V_{r}^{0}\rho_{r}-\alpha \rho_{r}V_{r}^{0}\left(1-\frac{\rho_{g}}{\rho_{r}}\right)$$

Another approach [8] considers that the decomposition of the material corresponds to a decrease in material density leading to the equation reported in Equation (2).

(2) $$\rho \left(t\right)=\rho_{v}-\overset{N}{\underset{i}{\sum }}\Delta \rho_{i}\alpha_{i}\;\left(t\right)\;\mathrm{with}\;0\leq \alpha_{i}\left(t\right)\leq 1,$$

where *t* is the time, *ρ*_v_, is the virgin density of the material, *α_i_*, the decomposition degree, and Δ*ρ*_i_, the total mass loss at each step *i.* In both methods, the expression of the density depends on the choice of the thermal decomposition model.

### 2.1. Kinetic Model

To characterise the decomposition degree, the decomposition kinetic parameters have to be determined. For that purpose, numerical models, namely “decomposition kinetic models”, were developed using a least-squares fitting procedure based on Thermogravimetric (TG) curves [9].

To identify the apparent kinetic decomposition model, TG measurements at various heating rates were performed [9,10]. The analyses of the decomposition mechanism permit to give a physical sense to the kinetic analysis. From a technical point of view, a kinetic analysis is also a tool for data reduction. From a series of measurements with many data points, a model with few parameters is extracted. The most known kinetic models to predict the TG-curves are the Friedman analysis [11], Ozawa–Flynn–Wall analysis [12], Kissinger analysis [13] and Coats–Redfern analysis [14]. No details are given on those different methods hereinafter [11,12,13,14,15,16,17,18] but we focus in the following section on kinetic parameters found by the authors for carbon fibre/epoxy composites. Depending on the number of decomposition step, single-step or multiple-step kinetic models were used. Table 1 summarises the value of the kinetic parameters found in the literature for carbon fibre/epoxy material considering various heating rates. 

#### 2.1.1. Single-Step Kinetic

A single-step kinetic model assumes that the apparent activation energy is constant during the decomposition process. The mass loss rate is then expressed using an Arrhenius function (Equation (3))

(3) $$\frac{\partial m}{\partial t}=-m_{0}{\left[\frac{m-m_{e}}{m_{0}}\right]}^{n}\mathrm{Aexp}\left(\frac{-E_{a}}{RT}\right),$$

where *T* is the temperature; *R* is the universal gas constant; *m* is the mass of the material; the subscripts 0 and *e* correspond to initial and final (end), respectively; *A* is the pre-exponential factor; *E_a_* is the apparent energy of activation; and *n* is the reaction order.

Quintiere et al. [21] selected a one-step first order decomposition model based on TG results carried out under nitrogen on a carbon fibre/epoxy composite. TG data were not consistent with the numerical results at high temperature, but the authors considered model as acceptable to predict the composite decomposition [21]. It suggests therefore that a single-step kinetic with a reaction order of 1 is not sufficient to describe with accuracy the kinetic of decomposition of a carbon fibre/epoxy composite. 

**Figure 1:**
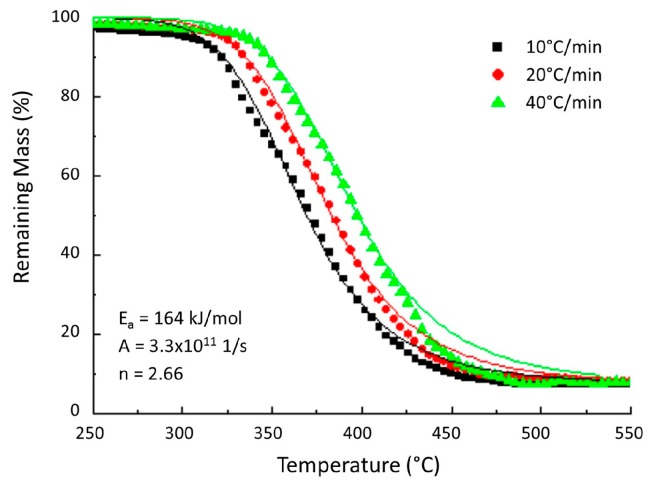
Comparison between the predicted and measured remaining mass based on Thermogravimetric results [21].

Burns et al. [22] simulated then TG curves of a woven carbon fibre/epoxy composite (Figure 1) by a one-step decomposition model.

In this study, the simulated curves are consistent with the numerical results in the whole temperature range. However, at the initial and final stages of the decomposition reaction, the simulated curves do not fit the experimental curves, probably because only one degradation stage was implemented in the model. Nevertheless, using the extracted kinetic parameters from the model, the authors gave a practical way to provide the time-to-failure of a carbon fibre/epoxy composite submitted to a fire-under-compression test. It suggests therefore that a simple kinetic model is sufficient to make thermomechanical model.

Another example is proposed by Chippendale et al. [20] (Table 1). They investigated the damage cause on T700/M21 when submitted to a lightning strike. The decomposition model is enough to simulate lightning strike (ablation phenomena prevail over decomposition phenomena) even if the simulated curves did not capture the whole decomposition of the composite.

#### 2.1.2. Multi-Step Kinetic

Sikoutris et al. [23] proposed a three-step decomposition model for carbon epoxy based composite. As shown in Table 1, the last step exhibits a reaction order superior to 3, which does not make sense. It suggests that the authors got a good agreement between numerical and experimental TG results because they fit the last step of the decomposition model. On the other hand, these authors proved that a better kinetic model improves slightly the modelling of the temperature at the rear face (this conclusion is only based on the first 40 s of the comparison between numerical and experimental fire test results).

To conclude on the kinetic modelling, decomposition models of the carbon fibre/epoxy composite reported in the literature were made to get the best fit between experimental and numerical weight loss curve. They do not discuss the physical meaning of the parameters and, hence, they cannot be used to build a real comprehensive model.

### 2.2. Thermal Properties

The measurement of temperature dependent-thermophysical properties of composite such as the anisotropic thermal conductivity and the specific heat capacity, is not easy, especially for high temperatures (>200 °C) [6]. Different methods are proposed in the literature to take into account the thermal properties such as the transverse thermal conductivity, *λ*_T_, and the isobar specific heat capacity, *C_p_*. Three methods are discussed in the following sections. 

**Figure 2:**
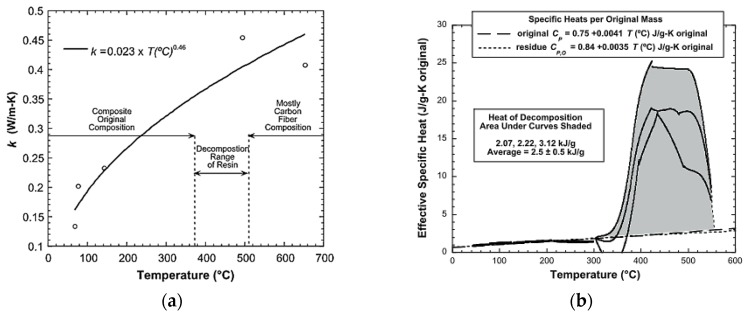
(**a**) Thermal conductivity versus temperature; and (**b**) specific heat capacity versus temperature and heat of decomposition of a carbon fibre/epoxy composite [19].

The first method is based on the measurement of the apparent properties of each state of material (virgin and final/degraded) as function of the temperature and/or time. Then, a mixture law (as Equation (4)), as a function of the decomposition degree or remaining mass, permits to find the best theoretical properties using empirical expressions. This approach was used by Quintiere et al. [21] presenting a homemade method (heater plate in specimen sandwich with edge insulation) to measure the thermal conductivity of a carbon fibre/epoxy composite. The authors considered an empirical expression for the thermal conductivity as a function of temperature (Figure 2a). 

They followed the same methodology to get the effective specific heat capacity (Figure 2b). The empirical expressions shown in Figure 2a,b were used by McGurn et al. [24] with the specific heat capacity also considered as a function of the decomposition degree (Equation (4)).

(4) $$C_{p}=\left(1-\alpha \right){C_{p}}_{0}\;+\;\alpha C_{p_{e}}$$

Other authors [25,26] carried out measurement on the virgin and decomposed materials (glass reinforced vinyl ester composite) to determine their thermal properties up to 800 °C but these methods cannot be applied to carbon epoxy laminates (different behaviour at high temperature).

The second method consists in the determination of the thermal properties of each constituent of the material (e.g., fibre, resin, carbonaceous residue (char), gas, and moisture) using known theoretical expression. Sikoutris et al. [23] calculated the thermal conductivity of composite, *λ*_comp_, using a homogenisation model (based on parallel thermal paths) including fibre, resin and gases (subscripts f, r and g) as a function of the decomposition degree, and of the initial volume fraction of resin and of fibre (Equation (5)).

(5) $$\frac{1}{\lambda_{\mathrm{comp}}}=\frac{V_{f}}{\lambda_{f}}\;+\;\left(1-\alpha \right)\frac{V_{r}^{0}}{\lambda_{r}}\;+\;\alpha \frac{V_{r}^{0}}{\lambda_{g}}$$

The authors considered that only the resin decomposes and that the mass of the resin is a function of the decomposition degree and of the initial resin content. The final resin mass is assumed to be zero and the volume fraction of gases is as a function of the decomposition degree and of the initial resin volume fraction. A strong assumption is that the gases fill totally the volume of the decomposed resin (no carbonaceous residue was considered). The authors considered also the heat of decomposition, *Q*_d_, in the expression of specific heat capacity of composite, *Cp*_comp_ (apparent specific heat capacity). In another paper [27], the authors proposed to express properties as a function of four components A, B, C and D and also as a function of the porosity. Unfortunately, this complexification did not bring any advantages.

The third method consists in determining the thermal properties using fitting methods [28,29,30] on the temperature and/or the mass of the sample measured during a totally controlled test (e.g., the Thermal Decomposition Apparatus (TDA) method [29]). More recently, an inverse method was also used to determine the thermal conductivity of a carbon fibre/epoxy laminates based on controlled experimental test [31]. For each stage, these properties are determined with least-squares difference between predicted and measured temperature. It is shown that estimations of material thermal properties are suitable assuming predictive models based on homogenisation theory.

Methods permit to determine the thermal conductivity and specific heat capacity versus temperature but only apparent values are obtained. One of the major issues is that, for some parameters, the measurement protocols are not completely established up to high temperature and the difficulty is to find an appropriate methodology. It is particularly true in the case of carbon fibre/epoxy composites. In this particular case, the thermal conductivity depends on the resin volume fraction, fibre volume fraction, inclusion volume fraction, shapes or arrangements of inclusions [32]. In the case of CFRP, the material is anisotropic. Some papers succeed to measure the tensor of thermal conductivity but only at low temperature (0–200 °C) [32] due to the apparition of cracks, thermal delamination and/or expansion after the temperature of decomposition. 

Considering the different approaches reported above, the first method is the most adequate for our study. Indeed, the second method requires knowing the properties of each constituent, which is not an easy task and often not all raw materials are available because they are proprietary. Moreover, when the material starts to degrade, it is difficult to measure the properties of each constituent as a function of the temperature. The last method (fitting procedure) is also not appropriate because the physical sense of the properties is lost.

To conclude, the determination of the density and/or decomposition degree is essential since the thermal properties, the heat capacity and the thermal conductivity are defined as a function of the temperature, time, density and/or decomposition degree. 

## 3. Experimental

### 3.1. Materials

T700/M21 carbon epoxy composite laminate was supplied by Airbus. This composite was manufactured using unidirectional prepregs with an isotropic stacking (ISO [0/45/90/135]s) or unidirectional stacking (UD [0]s). Details on the material are reported in a previous study [1]. The M21 resin is a complex formulation containing three types of epoxy resin (DiGlycidyl Ether Bisphenol F (DGEBF), TriGlycidylether meta-aminoPhenol (TGMAP) and Para-glycidyl amine), one hardener (4,4’-DiaminoDiphenyl Sulfone (DDS)) and thermoplastic nodules (PolyEther Sulfone (PES) and PolyAmide (PA6/PA12)) [33]. The fibres used in this composite are T700GC carbon fibres having high-resistance mechanical properties. The material studied was in virgin or degraded state. A pyrolysis was established to get a sample at a desired state of degradation. As the thermal decomposition of the material is known (see Section 4.2), the degree of decomposition of the material is known. Thus, the degraded state corresponds to a sample which reaches a thermally stable state composed of carbonaceous residue (yields from the resin decomposition) and fibre (inert or not).

### 3.2. Thermogravimetric Analysis

Thermogravimetric analysis (TGA) was carried out on TA Instruments (TA Q5000IR and TA Q600, TA Instruments, New Castle, DE, USA). The balance purge flow was set to 15 mL/min and the sample purge flow (nitrogen) to 50 mL/min. Thin square samples put in open alumina pan underwent a heating from 35 to 1100 °C (Q5000IR)/1500 °C (Q600) with a heating rate of 5, 10, 20, 50 and 100 °C/min. An isotherm of 45 min was performed before each measurement to ensure that atmosphere was inert.

### 3.3. Specific Heat Capacity

Specific heat capacity measurements were carried using differential scanning calorimetry (DSC) from TA Instruments (TA Q100, TA Instruments, New Castle, DE, USA). A modulated signal was used with a constant heating rate of 10 °C/min up 200 °C. The samples were crimped in alumina pan with lid. Additional results were obtained using a simultaneous thermal analysis from Netzsch (STA F1 Jupiter, Netzsch, Selb, Germany) to measure simultaneously the heat flow and the weight change of a sample with a constant modulated heating rate of 10 °C/min up to 1200 °C. The purge flow (nitrogen) was set to 100 mL/min. Thin square samples were put in closed platinum pan.

### 3.4. Thermal Diffusivity

A Light Flash Apparatus from Netzsch (LFA 467 HyperFlash, Netzsch, Selb, Germany) was used to measure the thermal diffusivity of the sample through-thickness and in-planes directions. During the experiments, a surface of the sample is heated by a Xenon flash lamp with variation of energy (by voltage and pulse-length). On the other surface of the sample, an infrared-detector measures the temperature increase to obtain the thermal diffusivity using optimisation methods.

### 3.5. Gas Phase Analysis

To characterise the gases released during fire testing, a Fourier Transform Infrared (FTIR) spectrometer was connected to fire tests. Thanks to this device, released gases are analysed online quantitatively and qualitatively. Gas picking system and transfer line were provided by M&C Tech Group (Ratingen, Germany). FTIR (AntarisTM Industrial Gas System) was provided by Thermo ScientificTM. (Waltham, MA, USA) The transfer line is 2 m long and maintained to a constant temperature of 180 °C using two temperature controllers. Two filters are placed on the line, one (2 μm) at the beginning of the gas sampling system, and another one (0.1 μm) placed in the middle of the line (at 1 m). The gas cell placed in the chamber of the spectrometer was set to 185 °C and 652 Torr. The optical pathway is 2 m long and the chamber of the spectrometer is filled with dry air. To quantify the gases, a methodology of quantification was performed using TQ Analyst software (V9.1.17, ThermoFisher Scientific Inc., Waltham, MA, USA). Spectra have to be collected for specific gases at different concentrations in the same conditions. These calibration spectra were provided by Thermo ScientificTM. The area under the peaks of characterised absorbance bands is linked with the concentration of released gases. Representative spectral regions for each gas were chosen to minimise interferences with other gases (see Section 4.6). Multidimensional interpolation models permit then to get a best fit of measured spectrum to provide a quantification of released gases.

The methods used to determine the properties of our composites are summarized in the Table 2.

## 4. Results and Discussion

### 4.1. Density

The density of virgin and degraded T700/M21 samples was determined by measuring the mass, the surface and the thickness of each sample using high precision balance and calliper. The virgin value obtained is 1575 kg/m^3^. It is close to the supplier value of 1580 kg/m^3^ [34]. For the degraded value, an assumption of the model is to assume that the volume is constant during the test. In that case, the final density is assumed to be at 1165 kg/m^3^, which correspond to a weight loss of 26 wt % of the 1575 kg/m^3^.

### 4.2. Kinetic Analysis

**Figure 3:**
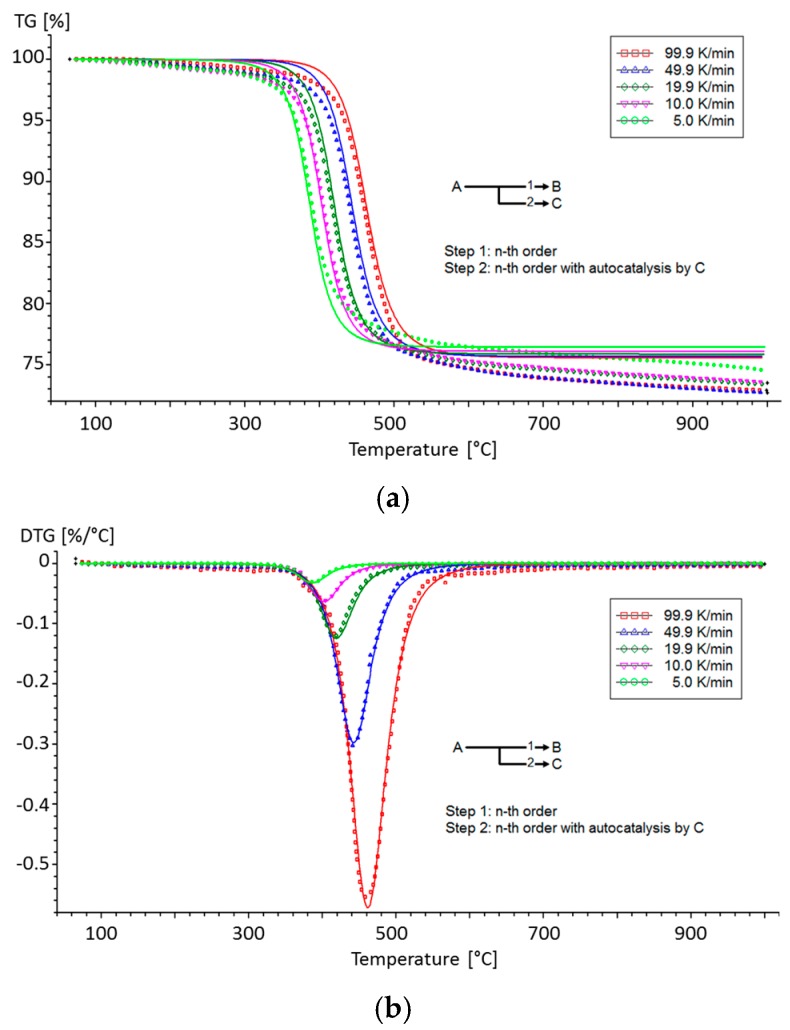
(**a**) Thermogravimetric curves and (**b**) differential thermogravimetric curves of the kinetic decomposition model with a physical meaning obtained using NETZSCH Thermokinetic software for T700/M21 [6].

The calculation of the kinetic parameters (the triplet: *E_a_, A,* and *n*) was made according to the method proposed by Moukhina [35] and detailed in a previous paper [6]. The gas phase analysis was also analysed taking into account the mechanism of decomposition of the material [6]. Figure 3 presents a comparison between the results obtained from TGA (TG and differential thermogravimetric curves) and from the determined kinetic decomposition model. 

The kinetic decomposition model is composed of two main competitive reactions (*n^th^* order and autocatalytic reactions) when T700/M21 decomposes under inert atmosphere. The corresponding kinetic parameters are presented in Table 3.

The main step of decomposition is an autocatalytic step with *E_a_* equal to 146 kJ/mol. This value is consistent with those reported in the literature for other epoxy resins. Indeed, the activation energy for the decomposition of DDS is reported to be equal to 142 ± 15 kJ/mol similar to that of the TGDDM/DDS (147 kJ/mol of 4,4′-Tetra-Glycidyl Diamino Diphenyl Methane) [36]. According to Li et al. [37], the activation energy of a carbon fibre/epoxy and a carbon fibre/epoxy/PES are similar and have values of about 120 kJ/mol. Thus, the PES does not change significantly the activation energy when added in an epoxy/CF composite. In addition, Rose [38] used the IKP (Invariant Kinetic Parameters) method to determine the parameters *E_a_* and *A* for the kinetic of decomposition of the TGDDM/DDS and found values of 125 kJ/mol.

The values obtained for the main step of decomposition (Step 2) are also consistent with the values previously reported in the literature for similar materials and close to the value reported for the DDS decomposition. Regarding Step 1, it is reported that changes in diluent (nodule and fortifiers) structure in the DGEBA-diluent blend modifies their thermal stability, and the activation energy of thermal degradation is in the range 48.3–84.5 kJ/mol [39]. Hence, the PA6 seems to influence the activation energy. For this reason, the first activation energy (58 kJ/mol) of Step 1 could be assigned to activation energy of the DGEBF included in the M21 with the PA6 blends.

### 4.3. Heat of Decomposition

**Figure 4:**
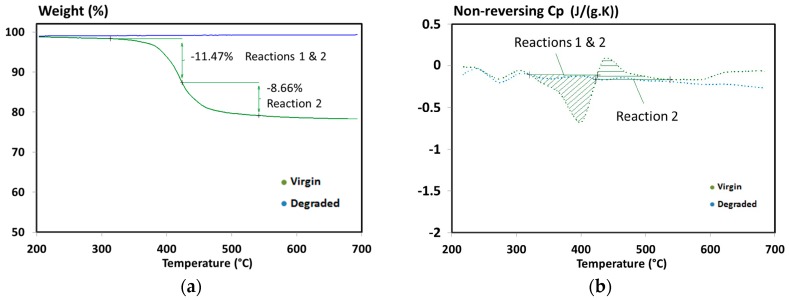
(**a**) Remaining mass; and (**b**) non-reversing specific heat capacity obtained during a simultaneous thermal analysis carried out under nitrogen to determine the heats of decomposition of T700/M21 [6].

The heats of decomposition were determined based on the modulated signal obtained using STA device. Modulated Differential Scanning Calorimetry (mDSC) measurements were performed instead of classical DSC to get a better accuracy and precision of the specific heat capacity. It permits to separate non-reproducible experimental artefacts from the measurement. The measurements under inert atmosphere were performed on a degraded sample to obtain a baseline, and on a virgin sample. Using both curves, heats of decomposition can be determined by integrating the area of decomposition peaks. Results of the non-reversing specific heat capacity and the remaining mass are presented in Figure 4. 

The first peak is an exothermic peak with a heat of decomposition of 2.5954 × 10^5^ J/kg (315 °C to 425 °C) and the second one is endothermic with a heat of decomposition of 1.5222 × 10^5^ J/kg (425 °C to 545 °C). It is noticed that the first peak is unusual because, generally, a decomposition of material is endothermic in inert atmosphere. These phenomena are assigned to several processes such as bond dissociation, new bond formation and gas evaporation. Some materials can produce an exothermic reaction under inert atmosphere [39]. Notably, PAN and bisphenol C polyarylate (extremely flame-resistant polymer) exhibit a high exothermic decomposition reaction. Most polymers with exothermic decomposition (under nitrogen) contain either halogens or some unsaturated double or triple bonds, which can lead to char formation by cross-linking or cyclization reactions.

This explanation can be linked to the decomposition mechanism of epoxy resins presented in previous works [6,40]. The decomposition occurs through cyclization of aliphatic chain ends and it explains the exothermic decomposition identified in our case. Similar results were recently reported by McKinnon et al. [31] where a carbon fibre/epoxy composite also exhibits exothermic and endothermic reactions. 

Other values were reported in the literature but these measurements were performed using standard Differential Scanning Calorimetry (DSC). As an example, only one exothermic heat of decomposition from 4 × 10^4^ J/kg to 1.4 × 10^5^ J/kg for a T700/M21 composite (large peak around 390 °C) was found [20]. These values are quite different from our values but they are questionable according to the measurement and data treatment methods. In our case, a more precise method was used since the mass loss of the sample was measured simultaneously and a standard DSC cannot be as precise as modulated DSC (mDSC) when a material decomposes.

### 4.4. Effective Specific Heat Capacity

**Figure 5:**
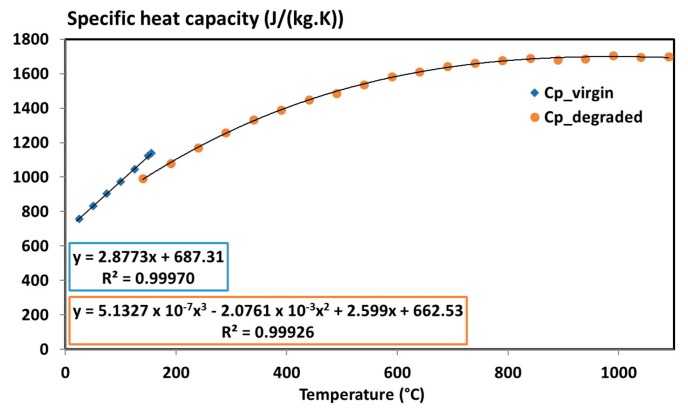
Specific heat capacity of the virgin and degraded materials obtained with DSC Q100 and STA F1 measurements, respectively [6].

The specific heat capacity of the virgin composite was measured with mDSC up to 200 °C (before any decomposition reaction). Measurements on degraded materials were performed with dynamic modulated method up to 1100 °C. As far as we know, it is the first time such method is applied on an anisotropic carbon fibre/epoxy composite. The reversible signal measurements obtained on the virgin and degraded materials are presented in Figure 5. 

In both cases, an increasing specific heat capacity with increasing temperature is observed. The specific heat capacity of the non-degraded sample is close to the values found in the literature for similar materials [30,32,41]. The degraded sample shows an increase of its specific heat capacity up to 1600 J/(kg·K) at 1000 °C. It was reported [42] that the specific heat capacity of carbon fibre/carbon composite can reach a value of 2020 J/(kg·K) at 1230 °C. To check the measurements on the degraded material, a measurement on T700 fibre alone using standard DSC up to 400 °C is also performed, and similar values as those of the degraded material are found. Hence, the measured values for both material states (virgin and degraded) are accurate and can be used as input data in a model. A linear segment is considered for the virgin state up to 200 °C and a three-degree polynomial expression is considered for the degraded state. The specific heat capacity of T700/M21 is expressed as a function of these two temperature-dependent interpolations and of the decomposition degree using a mixture law as presented in Equation (4).

### 4.5. Effective Thermal Conductivity

The thermal diffusivity was measured up to 450 °C on virgin and degraded materials. As discussed by Tranchard et al. [1], the composite has an anisotropic structure [32,43] and so, measurements in the three main directions of the composite is needed.

**Figure 6:**
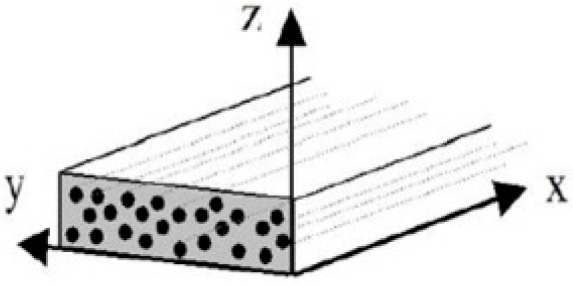
Coordinate system associated to an unidirectional composite [31].

The orthogonal coordinate system (*Oxyz*) associated to an UD composite is represented in Figure 6. The through-thickness direction corresponds to the thickness direction of the coupon (*Oz*) and to the stacking direction of the plies. The two in-plane directions correspond to (*Ox*) and to (*Oy*). In the case of UD composite, (*Oy*) and (*Oz*) are perpendicular to the fibre direction (*Ox*) (0° orientation of the fibre in a layer). These three directions are the main directions of the composite and correspond to the diagonal terms in the diffusivity tensor, *A* (Equations (6) and (7)).

Thermal diffusivity measurements were performed on a virgin, *v*, and degraded, *e*, material. Based on these measurements and those of the density and of the specific heat capacity, the thermal conductivity tensor, *Λ*, of both states of material can be calculated (Equation (8)).

(6) $$A_{v}=\left[\begin{array}{ccc} a_{v_{x}} & 0 & 0\\ 0 & a_{v_{y}} & 0\\ 0 & 0 & a_{v_{z}} \end{array}\right]\;,$$

(7) $$A_{d}=\left[\begin{array}{ccc} a_{d_{x}} & 0 & 0\\ 0 & a_{d_{y}} & 0\\ 0 & 0 & a_{d_{z}} \end{array}\right],$$

(8) $$\Lambda_{v_{i}}=A_{v_{i}}\rho_{v}C_{p_{v}}\;;\;\Lambda_{e_{i}}=A_{e_{i}}\rho_{e}C_{p_{e}}\;,$$

Based on the homogenisation strategy from the literature [32,44] the effective thermal conductivity tensor of a composite material is expressed as a function of the each *i^th^* ply thermal conductivity and its orientation, *θ_i_*, and thickness, *d_i_*. Consequently, each layer tensor was calculated for each orientation for both stacking (ISO and UD) to obtain the effective thermal conductivity tensor of the composite material in the coordinate system of the composite (*O, x, y, z*).

In the case of the ISO composite, the effective thermal conductivity of the virgin and degraded samples is expressed according to Equations (9)–(11) taking into account the assumption of homogeneous interlayers:

(9) $${\Lambda_{c}}_{ISO}=\left[\begin{array}{ccc} \frac{1}{2}\left(\lambda_{x_{x}}\;+\;\lambda_{y_{y}}\right) & 0 & 0\\ 0 & \frac{1}{2}\left(\lambda_{x_{x}}\;+\;\lambda_{y_{y}}\right) & 0\\ 0 & 0 & \lambda_{z_{z}} \end{array}\right],$$

where

(10) $$\lambda_{v_{x}}=\;\lambda_{v_{y}}=\frac{1}{2}{\left(\lambda_{x_{x}}+\lambda_{y_{y}}\right)}_{v}=\;\lambda_{v_{x_{\mathrm{measured}}}}$$

and

(11) $$\lambda_{d_{x}}=\;\lambda_{d_{y}}=\frac{1}{2}{\left(\lambda_{x_{x}}\;+\;\lambda_{y_{y}}\right)}_{d}=\;\lambda_{d_{x\_\mathrm{measured}}}$$

Thus, *λ_vx_* and *λ_vy_* are equal for the ISO composite. Then, in the case of the UD composite, each layer tensor is equal (only one orientation, 0°). Therefore, the effective thermal conductivity tensor is expressed by Equation (12) taking into account the assumption of homogeneous interlayers.

(12) $${\Lambda_{c}}_{UD}=\left[\begin{array}{ccc} \lambda_{x_{x}} & 0 & 0\\ 0 & \lambda_{y_{y}} & 0\\ 0 & 0 & \lambda_{z_{z}} \end{array}\right]$$

An additional assumption is to consider the equality between the (*Oy*) and (*Oz*) direction for the UD composite. It is then possible to deduce the UD tensor from the ISO sensor (Equations (13)–(15)).

(13) $${\Lambda_{c}}_{UD}=\left[\begin{array}{ccc} \lambda_{x_{x}} & 0 & 0\\ 0 & \lambda_{z_{z}} & 0\\ 0 & 0 & \lambda_{z_{z}} \end{array}\right],$$

where

(14) $$\lambda_{v_{x_{\mathrm{measured}}}}=\;\frac{1}{2}\left(\lambda_{x_{x}}\;+\;\lambda_{y_{y}}\right)\;and\;\lambda_{y_{y}}=\lambda_{z_{z}}=\lambda_{z_{z\_\mathrm{mesured}}}$$

Thus,

(15) $$\lambda_{x_{x}}=2\;\lambda_{v_{x\_\mathrm{measured}}}-\lambda_{z_{z\_\mathrm{mesured}}}$$

The in-plane thermal conductivities in (*Ox*) and (*Oy*) directions are the same for an ISO composite and only one measurement in-plane direction is thus necessary.

**Figure 7:**
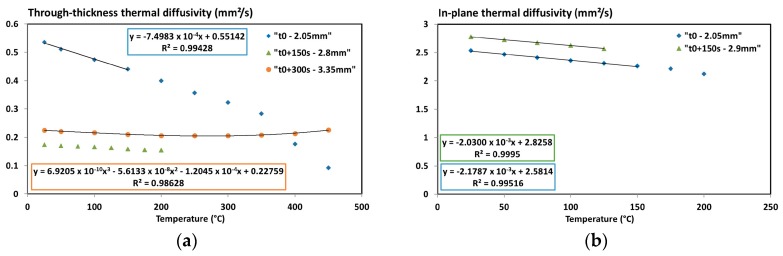
(**a**) Through-thickness; and (**b**) In-plane thermal diffusivities of the T700/M21 composite (virgin, half-degraded and degraded states) as a function of the temperature [6].

The through-thickness thermal diffusivity of a virgin coupon (written *t*0 in the caption) and two degraded coupons (*t*0 + 150 s; *t*0 + 300 s) as a function of temperature were measured (Figure 7a). Coupons were exposed to the fire test developed by Tranchard et al. [1] for 150 s and 300 s to get a measurement as a function of the decomposition degree, i.e., half-degraded and degraded. The thicknesses of each coupon are indicated in the caption of Figure 7.

The thermal diffusivity of the virgin material decreases linearly up to 350 °C and then the slope of the curve becomes steeper. The temperature of 350 °C is close to the temperature of decomposition of the composite. The formation of cracks and thermal delamination increase the thickness of the sample (considered as constant during the measurements) and hence the measured thermal diffusivity decreases. The half-degraded and degraded samples show a much lower thermal diffusivity at low temperature, and then a weaker dependence to the temperature. Both degraded materials have quite different thermal diffusivity due to their different thermal history. The thermal diffusivity of the degraded sample increases slightly above 300 °C, which is due to further structural changes and increasing radiation within the sample. At high temperatures (>400 °C), the virgin sample has 2–3 times lower thermal diffusivity than the burnt material. This difference can be explained by the different thicknesses of material, decomposition state.

The in-plane thermal diffusivity of the virgin and half-degraded samples are measured up to 250 °C (it was difficult to cut sample strips on degraded coupons and thus measurement on the sample completely degraded was not possible). The results differ from the results obtained from the transvers measurement (Figure 7b). Only one direction (*Oy*) was measured on the ISO composite since the thermal conductivity following *Ox* and *Oy* are the same (symmetric stacking). The results show that the in-plane thermal diffusivity is 10 times higher than through plane (Figure 7). It confirms the thermal anisotropy of this material. Small differences between the virgin and half-degraded samples were detected. It is assigned to heat transfer in horizontal direction, which is more or less independent from the decomposition degree (but more investigations are necessary). On the other hand, the virgin and half-degraded materials keep the same mass fraction of fibre (they are not degraded [1]) but they have different mass fraction of resin and of carbonaceous residue. At the end, two linear segments are considered for the modelling of the virgin state.

**Figure 8:**
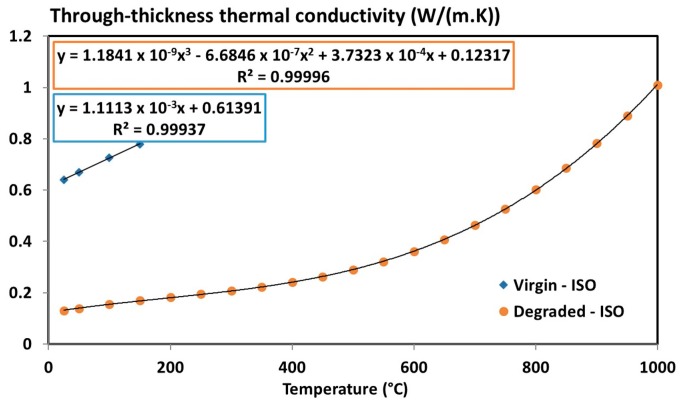
Through-thickness thermal conductivity of the T700/M21 composite (virgin and degraded state) as a function of the temperature [6].

Therefore, only the virgin and final states of material are necessary to express the thermal conductivity tensor of T700/M21 using a mixture law (as Equations (4) and (8)). First, the through-thickness thermal conductivity can be determined (Figure 8) based on the determination of the thermal diffusivity, specific heat capacity and density of the virgin and degraded materials (Equation (8)).

A linear segment is considered for the virgin state up to 150 °C. A three-degree polynomial expression is considered for the degraded state. This behaviour is consistent with the literature since it is reported that the thermal conductivity of degraded material varies with a third power of temperature (for the through-thickness only) due to the pores present inside the material which generates internal radiation [44,45]. Generally, this third power term is given as a function of the pore diameter and of the radiative exchange factor. The higher the size of pore, the greater must be the radiation inside the porous network [44,45]. 

**Figure 9:**
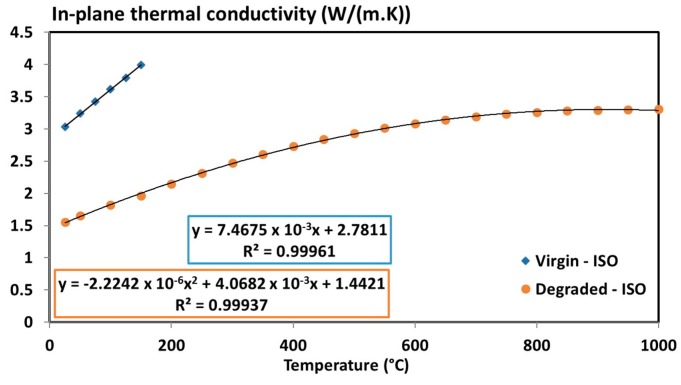
In-plane thermal conductivity of the T700/M21 composite (virgin and degraded state) as a function of the temperature [6].

The in-plane thermal conductivities of the virgin and degraded states are presented in Figure 9. The results show that the T700/M21 exhibits an anisotropic behaviour with an in-plane thermal conductivity 11 times higher than the through-thickness thermal conductivity before 500 °C and six times higher after 500 °C. As evidenced previously [32,46], a factor of 8 can be observed between the through-thickness and in-plane thermal conductivity. These results are thus consistent with the literature data. A linear variation of the thermal conductivity of the virgin material up to 200 °C and a second degree polynomial expression for the degraded material is obtained. 

**Figure 10:**
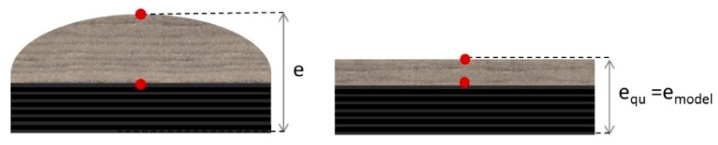
Scheme of the determination of the equivalent thermal conductivity with a volume constant assumption [6].

As discussed previously, it was assumed that the volume is constant during the test (assumption for further modelling). In that case, the thermal conductivity of degraded material previously determined have to be updated. An equivalent conductance, *C*_th_, is used to determine the apparent thermal conductivity at degraded state, *λ*_equ_, taking into account the thermal expansion of the material. Expanded or not, the same heat flux goes through the material (Figure 10, Equations (16) and (17)). Thus, considering the same surface, *S*, the expression of the apparent thermal conductivity is described as follows.

(16) $$C_{\mathrm{th}}=\frac{\lambda }{e}\;S=\frac{\lambda_{\mathrm{equ}}}{e_{\mathrm{equ}}}S_{\mathrm{equ}}\;,$$

where

(17) $$S=S_{\mathrm{equ}}\;and\;\lambda_{\mathrm{equ}}=\frac{e_{\mathrm{equ}}}{e}\lambda$$

**Figure 11:**
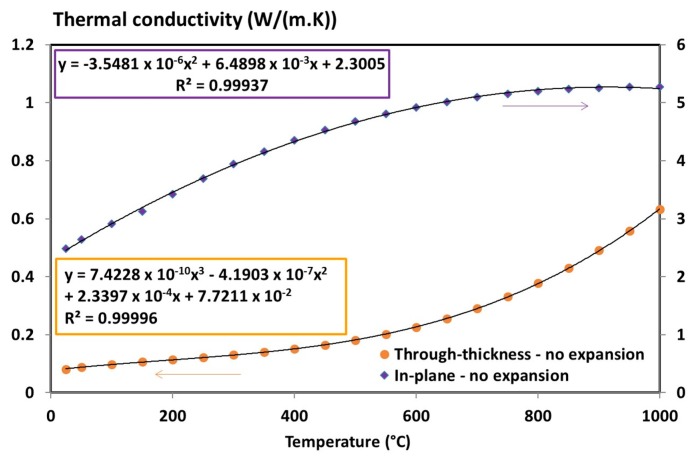
Through-thickness and in-plane thermal conductivity of the degraded T700/M21 composite as a function of the temperature [6].

Hence, the final thermal conductivity tensor determined previously have to be multiplied by the ratio 2.1/3.35 in through-thickness direction (the thickness of the sample divided by the “without-expansion” thickness of the coupons, *e*_equ_), and divided by its ratio in longitudinal direction. Figure 11 shows the updated of the interpolation curves with thermal expansion taken into account in the thermal conductivity expressions. 

### 4.6. Specific Heat Capacity of Gases

The quantity of main released gases was measured during a controlled fire test coupled with a Fourier transform infrared spectrometer (FTIR). It is a Mass loss cone calorimeter (ISO13927 [47,48]) which permits to perform measurement under pyrolytic conditions (less than 2% of oxygen) at an external heat flux of 50 kW/m^2^ [6]. It is proven that the decomposition of the T700/M21 is similar with this low amount of oxygen than under full nitrogen atmosphere. 

When the quantity of each gas is measured, a mixture law can be applied as a function of mass fraction of main gases. Then, it is possible to determine the specific heat capacity of gases knowing thermophysical property of each gases (tables available in the literature). 

A quantification methodology taking into account the possible gases released from the decomposition of the T700/M21 and the available calibration gases (H_2_O, CO_2_, CO, NO, NO_2_, N_2_O, NH_3_, SO_2_, HCN, CH_4_, C_2_H_2_, C_2_H_4_, C_3_H_6_, C_3_H_8_, C_3_H_4_O, CH_2_ and CH_3_COOH) was developed [1]. This methodology is based on the work of Marker and Speitel [49]. 

Through the FTIR analyses, the average volume fraction of gases released during the decomposition of the T700/M21 was identified. The ratio of each main gas was calculated using the quantity of gas released at each time divided by the total quantity measured. An average ratio is then determined and presented in Table 4. It is observed that the main gases released are water and carbon dioxide. Nevertheless, the small quantity of methane, carbon dioxide, sulphur dioxide and ammonia is sufficient to influence the gas properties. Other gases such as phenol and carbonyl sulphide are detected but they are neglected because of their low quantity.

Based on the average volume fraction of main gas released, an average isobar specific heat of gases can be determined as a function of the temperature. Tables from NIST database [49] permit to get the density, *ρ_i_*, and the specific heat capacity, *C_p__g,i_*, of each main gas released. The law of mixture of each gas can be used as a function of their mass fraction, *W_i_*, and of their specific heat capacity (Equation (18)).

(18) $${C_{p}}_{g}=\;\underset{i}{\sum }C_{p_{g_{i}}}W_{i}=\;\frac{1}{\rho_{g}}\underset{i}{\sum }C_{p_{g_{i}}}\rho_{i}V_{i}\;$$

**Figure 12:**
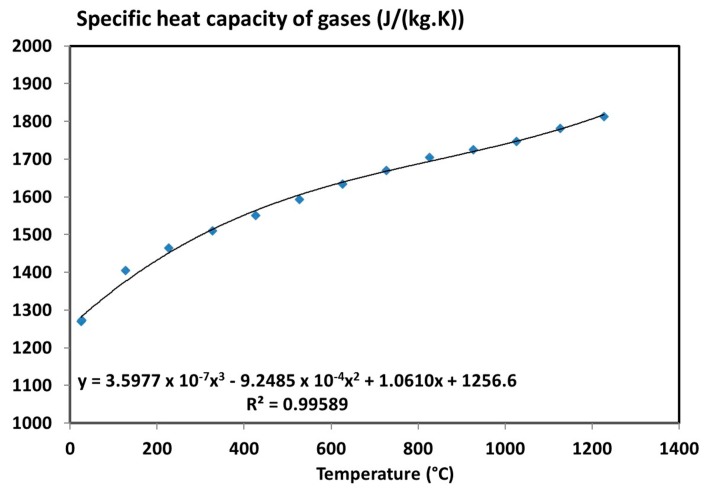
Average isobar specific heat capacity of gases released during the decomposition of the T700/M21 as a function of the temperature [6].

The specific heat capacity of gases was then calculated as a function of the temperature (Figure 12). It can be expressed using a third-degree polynomial interpolation (as commonly used for specific heat of gases in literature [50]).

### 4.7. Summary

The properties measured in this paper can provide input data for further modelling of carbon fibre/epoxy composite in fire (see Part II of this paper). 

All inputs determined in this paper were used in the 3D modelling of the T700/M21 composite and they are summarized in Table 5. As a bonus, this table offers a public database of input data necessary to model fire behaviour of an aircraft carbon fibre/epoxy composite laminate.

## 5. Conclusions

Thermal and physical properties of T700/M21 composite were determined from ambient up to high temperature (1000 °C) using thermogravimetric analysis, simultaneous thermal analysis, laser flash analysis, and Fourier transform infrared spectrometer. Thermal properties such as anisotropic thermal conductivity and specific heat capacity of material were determined as a function of the temperature and of the decomposition degree. Specific heat capacity of gases, heat of decomposition, kinetic decomposition model, and density of material were measured and were fully discussed. All of these properties and the associated protocols permit to create a public database necessary to provide full characterisation of a carbon fibre/epoxy composite laminate exposed to fire or to high temperature. These properties are specific to a type of carbon fibre/epoxy resin, i.e., carbon epoxy laminates applied to aircraft structure. As a bonus, inputs for further modelling with a real physical sense were obtained creating a unique database for fire safety engineering.

## Figures and Tables

**Table 1:** Kinetic parameters of different carbon epoxy materials based on TG analysis under nitrogen *n^th^* order decomposition kinetic model.

Material	Step	Heating Rate (°C/min)	*n* (-)	*A* (1/s)	*E_a_* (kJ/mol)	
Carbon fibre/epoxy	1	1, 3, 10, 30	1	9.67 × 10^10^	182	[19]
Woven carbon fibre/R118	1	10, 20, 40	2.68	3.3 × 10^11^	164	[18]
T700/M21	1	5, 10, 20	1.344	3.15 × 10^11^	181.73	[20]
Carbon fibre/epoxy	1	10–50	1.272	2.14 × 10^10^	165.432	[21]
	2		1.902	54.2	60.472	
	3		3.651	3111.22	36.236	

**Table 2:** Equipment used to determine the input properties necessary for modelling.

Properties		Equipment Used
Names	Unit	
Virgin density, $\rho_{0}=\frac{m_{0}}{V}$	(kg/m^3^)	High precision balance + calliper
Degraded density, $\rho_{e}=\frac{m_{e}}{V}$	(kg/m^3^)	High precision balance + calliper
Arrhenius parameters, $A_{i},\;E_{i},\;n_{i}$	(1/s, J/mol, -)	Kinetic analysis based on numerical methods
Virgin specific heat capacity, $\;C_{p_{v}}$	(J/(kg·K))	Modulated DSC up to the decomposition temperature, reversing signal
Degraded specific heat capacity, $C_{p_{e}}$	(J/(kg·K))	Modulated DSC up to 1100 °C, reversing signal
Heats of decomposition, $Q_{d_{i}}$	(J/kg)	Modulated DSC up to 1100 °C, non-reversing signal
Tensor of the virgin thermal conductivity, $\Lambda_{v}$	(W/(m·K))	Measurement of the thermal diffusivity using LFA in the three main directions
Tensor of the degraded thermal conductivity, $\Lambda_{e}$	(W/(m·K))	Measurement of the thermal diffusivity using LFA in the three main directions

**Table 3:** Kinetic parameters determined for the decomposition of the T700/M21 composite.

Reaction		Step 1	Step 2	Unit
*n^th^* order decomposition				
Activation energy	*E_a_*	58.7107	146.6740	(kJ/mol)
Frequency	Log *A*	0.8585	8.7268	(1/s)
Order	*n*	1.1125	2.0825	(-)
Contribution	Fraction	0.5540	0.9479	(-)
Autocatalysis				
Constant	log*K*_cat_	-	0.8744	(-)

**Table 4:** Average volume fraction of main gas released when T700/M21 is exposed to controlled radiative heat flux of 50 kW/m² under nitrogen atmosphere.

Gases	H_2_O	CO_2_	CO	SO_2_	CH_4_	NH_3_
Volume fraction (*V_i_*) (-)	0.675	0.213	0.038	0.039	0.019	0.01

**Table 5:** Input data based on experimental tests on the T700/M21 composite [6]

Name	Exp.	Value ^1^	Unit
Virgin density	$\rho_{0}$	1575	(kg/m^3^)
Degraded density	$\rho_{e}$	1165	(kg/m^3^)
Virgin specific heat capacity	$C_{p_{v}}$	2.8773 *T* + 687.31 with 20 °C ≤ *T* ≤ 300 °C	(J/(kg·K))
Degraded specific heat capacity	$C_{p_{e}}$	5.1327 × 10^−7^ *T*^3^ − 2.0761 × 10^−3^ *T*^2^ + 2.599 *T* + 662.53	(J/(kg·K))
Gas specific heat capacity	$C_{p_{g}}$	3.5977 × 10^−7^ *T*^3^ − 9.2485 × 10^−4^ *T*^2^ + 1.0610 *T* + 1256.6	(J/(kg·K))
Tensor of the virgin thermal conductivity, $\Lambda_{v}$	$\lambda_{v//}$	7.4675 × 10^−3^ *T* + 2.7811 with 20 °C ≤ *T* ≤ 150 °C	(W/(m·K))
	$\lambda_{v⊥}$	1.1113 × 10^−3^ *T* + 0.61391 with 20 °C ≤ *T* ≤ 150 °C	(W/(m·K))
Tensor of the thermal conductivity, $\Lambda_{v}$	$\lambda_{d//}$	−3.5481 × 10^−6^ *T*^2^ + 6.4898 × 10^−3^ *T* + 2.3005	(W/(m·K))
	$\lambda_{d⊥}$	7.4228 × 10^−10^ *T*^3^ − 4.1903 × 10^−7^ *T*^2^ + 2.3397 × 10^-4^ *T* + 7.7211 × 10^−2^	(W/(m·K))
Heats of decomposition	$Q_{d_{1}}$	259.54	(kJ/kg)
	$Q_{d_{2}}$	−152.22	(kJ/kg)
Arrhenius parameters step 1	$A_{1},$ $\;E_{1},\;n_{1},\chi_{1}$	7.2193; 58.7107; 1.1125; 0.554	(1/s, kJ/mol, -, -)
Arrhenius parameters step 2	$A_{2},\;$ $E_{2},\;n_{2},$ $K_{cat2},\;\chi_{2}$	5.3309 × 10^8^; 146.674; 2.0825; 7.4886; 0.9479	(1/s, kJ/mol, -) (-, -)

^1^ with temperature in °C.

## References

[1] Tranchard P., Samyn F., Duquesne S., Thomas M., Estèbe B., Montès J.-L., Bourbigot S. (2015). Fire behaviour of carbon fibre epoxy composite for aircraft: Novel test bench and experimental study. J. Fire Sci..

[2] ISO2685:1998(E) (1998). Aircraft—Environmental Test Procedure for Airborne Equipment—Resistance to Fire in Designated Fire Zones.

[3] Gibson A.G., Mouritz A.P., Wu Y., Gardiner C.P., Mathys Z. (2003). Validation of the gibson model for the fire reaction properties of fibre-polymer composites. Composites in Fire 3: 3rd International Conference on the Response of Composite Materials to Fire.

[4] Gibson A.G., Wright P.N.H., Wu Y.-S., Mouritz A.P., Mathys Z., Gardiner C.P. (2004). The integrity of polymer composites during and after fire. J. Compos. Mater..

[5] Tranchard P., Thomas M. (2012). Modélisation de la thermo-dégradation d’un composite aéronautique. Congrès Français de Thermique.

[6] Tranchard P. (2015). Modelling the Behaviour of a Carbon/Epoxy Composite Submitted to Fire. Ph.D. Thesis.

[7] Pering G.A., Farrell P.V., Springer G.S. (1980). Degradation of tensile and shear properties of composites exposed to fire or high temperature. J. Compos. Mater..

[8] Eekelen A.J., Bouilly J.M., Hudrisier S., Aspa Y. (2009). Design and Numerical Modelling of Charring Material Ablators for Re-Entry Applications.

[9] Nelson J.B. (1967). Determination of Kinetic Parameters of Six Ablation Polymers by Thermogravimetric Analysis.

[10] Mamleev V., Bourbigot S., Bras M.L., Lefebvre J. (2004). Three model-free methods for calculation of activation energy in tg. J. Therm. Anal. Calorim..

[11] Friedman H.L. (1964). Kinetics of thermal degradation of char-forming plastics from thermogravimetry. application to a phenolic plastic. J. Polym. Sci. Part C Polym. Symp..

[12] Ozawa T. (1965). A new method of analyzing thermogravimetric data. Bull. Chem. Soc. Jpn..

[13] ASTM_E698 (2016). Standard Test Method for Arrhenius Kinetic Constants for Thermally Unstable Materials Using Differential Scanning Calorimetry and the Flynn/Wall/Ozawa Method.

[14] Coats A.W., Redfern J.P. (1964). Kinetic parameters from thermogravimetric data. Nature.

[15] Flynn J.H. (1983). The isoconversional method for determination of energy of activation at constant heating rates. J. Therm. Anal..

[16] Opfermann J., Kaisersberger E. (1992). An advantageous variant of the ozawa-flynn-wall analysis. Thermochim. Acta.

[17] Brown M.E., Maciejewski M., Vyazovkin S., Nomen R., Sempere J., Burnham A., Opfermann J., Strey R., Anderson H.L., Kemmler A. (2000). Computational aspects of kinetic analysis: Part a: The ictac kinetics project-data, methods and results. Thermochim. Acta.

[18] Šesták J., Berggren G. (1971). Study of the kinetics of the mechanism of solid-state reactions at increasing temperatures. Thermochim. Acta.

[19] ASTM_E176 (2010). Standard Terminology of Fire Standards.

[20] Chippendale R.D., Golosnoy I.O., Lewin P.L. (2014). Numerical modelling of thermal decomposition processes and associated damage in carbon fibre composites. J. Phys. D Appl. Phys..

[21] Quintiere J.G., Walters R.N., Crowley S. (2007). Flammability Properties of Aircraft Carbon-Fiber Strutural Composite.

[22] Burns L.A., Feih S., Mouritz P.A. (2009). Fire-Under-Load Testing of Carbon Epoxy Composites.

[23] Sikoutris D.E., Vlachos D.E., Kostopoulos V. (2011). Multi stage decomposition modeling of polymer matrix composites. Composites in Fire 6, 6th International Conference on Composites in Fire.

[24] McGurn M.T., DesJardin P.E., Dodd A.B. (2012). Numerical simulation of expansion and charring of carbon-epoxy laminates in fore environments. Int. J. Heat Mass Transf..

[25] Lattimer B.Y., Ouellette J., Trelles J. (2009). Thermal response of composite materials to elevated temperatures. Fire Technol..

[26] Lattimer B.Y., Ouellette J. (2006). Properties of composite materials for thermal analysis involving fires. Compos. A Appl. Sci. Manuf..

[27] Sikoutris D.E., Vlachos D.E., Kostopoulos V., Jagger S., Ledin S. (2011). Fire burnthrough response of cfrp aerostructures. Numerical investigation and experimental verification. Appl. Compos. Mater..

[28] Bai Y. (2009). Material and Structural Performance of Fiber-Reinforced Polymer Composites at Elevated and High Temperatures. Ph.D. Thesis.

[29] Lattimer B.Y., Ouellette J., Trelles J. (2011). Measuring properties for material decomposition modeling. Fire Mater..

[30] Scott E., Beck J.V. (1992). Estimation of thermal properties in epoxy matrix/carbon fiber composite materials. J. Compos. Mater..

[31] McKinnon M., Stoliarov S. (2015). Pyrolysis model development for a multilayer floor covering. Materials.

[32] Thomas M. (2008). Proprietes Thermiques de Materiaux Composites: Caracterisation Experimentale et Approche Microstructurale. Ph.D. Thesis.

[33] Paris C., Bernhart G., Olivier P.A., De Almeida O. (2012). Influence de cycles de cuisson rapides sur le préimprégné aéronautique M21/T700: Suivi de polymérisation et propriétés mécaniques. Matériaux Techniques.

[34] Hexcel Hexply^®^ M21 Epoxy Resin. http://www.aerosparesltd.com/files/hexcel/hexply_m21.pdf.

[35] Moukhina E. (2012). Determination of kinetic mechanisms for reactions measured with thermoanalytical instruments. J. Therm. Anal. Calorim..

[36] Buch J.D. (1982). Thermal Expansion Behavior of a Thermally Degrading Organic Matrix Composite.

[37] Li C., Kang N., Labrandero S.D., Wan J., González C., Wang D. (2014). Synergistic effect of carbon nanotube and polyethersulfone on flame retardancy of carbon fiber reinforced epoxy composites. Ind. Eng. Chem. Res..

[38] Rose N. (1995). Thermal Degradation and Fire Performance of Epoxy Resins Used in the Aeronautic Industry. Ph.D. Thesis.

[39] Zhang H. (2004). Fire-Safe Polymers and Polymer Composites.

[40] Levchik S.V., Weil E.D. (2004). Thermal decomposition, combustion and flame-retardancy of epoxy resins—A review of the recent literature. Polym. Int..

[41] Kalogiannakis G., Van Hemelrijck D., Van Assche G. (2004). Measurements of thermal properties of carbon/epoxy and glass/epoxy using modulated temperature differential scanning calorimetry. J. Compos. Mater..

[42] Cezairliyan A., Miiller A.P. (1980). Specific heat capacity and electrical resistivity of a carbon-carbon composite in the range 1500–3000 k by a pulse heating method. Int. J. Thermophys..

[43] Lorette C. (2007). Outils de Caractérisation Thermophysique et Modèles Numériques pour les Composites Thermostructuraux à Haute Température. Ph.D. Thesis.

[44] Henderson J.B., Wiebelt J.A., Tant M.R. (1985). A model for the thermal response of polymer composite materials with experimental verification. J. Compos. Mater..

[45] Henderson J.B., Wiecek T.E. (1987). A mathematical model to predict the thermal response of decomposing, expanding polymer composites. J. Compos. Mater..

[46] Mouritz A.P., Gibson A.G. (2006). Fire Properties of Polymer Composite Materials.

[47] Babrauskas V. (2008). The cone calorimeter. The SFPE Handbook of Fire Protection Engineering.

[48] ISO13927 (2001). Simple Heat Release Test Using a Conical Radiant Heater and a Thermopile Detector.

[49] Marker T.R., Speitel L.C. (2008). Development of a Laboratory-Scale Test for Evaluating the Decomposition Products Generated inside an Intact Fuselage during a Simulated Postcrash Fuel Fire.

[50] Lemmon E.W., McLinden M.O., Friend D.G., Linstrom P.J., Mallard W.G. (1998). Thermophysical Properties of Fluid Systems in NIST Chemistry WebBook.

